# Cytomegalovirus Exposure and the Risk of Overall Infection After Kidney Transplantation: A Cohort Study on the Indirect Effects Attributable to Viral Replication

**DOI:** 10.3389/ti.2021.10273

**Published:** 2022-01-20

**Authors:** Isabel Rodríguez-Goncer, María Ruiz-Ruigómez, Francisco López-Medrano, Hernando Trujillo, Esther González, Natalia Polanco, Eduardo Gutiérrez, Rafael San Juan, Laura Corbella, Tamara Ruiz-Merlo, Patricia Parra, María Dolores Folgueira, Amado Andrés, José María Aguado, Mario Fernández-Ruiz

**Affiliations:** ^1^ Unit of Infectious Diseases, Hospital Universitario “12 de Octubre”, Instituto de Investigación Sanitaria Hospital “12 de Octubre” (imas12), Madrid, Spain; ^2^ Centro de Investigación Biomédica en Red (CIBER) de Enfermedades Infecciosas, Madrid, Spain; ^3^ School of Medicine, Universidad Complutense, Madrid, Spain; ^4^ Department of Nephrology, Hospital Universitario “12 de Octubre”, Instituto de Investigación Sanitaria Hospital “12 de Octubre” (imas12), Madrid, Spain; ^5^ Department of Microbiology, Hospital Universitario “12 de Octubre”, Instituto de Investigación Sanitaria Hospital “12 de Octubre” (imas12), Madrid, Spain

**Keywords:** cytomegalovirus, kidney transplantation, indirect effects, infection, opportunistic infection

## Abstract

Previous reports hypothesized that cytomegalovirus (CMV) may predispose to non-CMV infection after kidney transplantation (KT). We analysed the incidence of non-CMV infection (overall, bacterial and opportunistic) in 291 KT recipients according to the previous development of any level or high-level (≥1,000 IU/ml) CMV viremia. Exposure to CMV replication was assessed throughout fixed intervals covering first the 30, 90, 180 and 360 post-transplant days (cumulative exposure) and non-overlapping preceding periods (recent exposure). Adjusted Cox models were constructed for each landmark analysis. Overall, 67.7 and 50.5% patients experienced non-CMV and CMV infection, respectively. Patients with cumulative CMV exposure had higher incidence of non-CMV infection beyond days 30 (*p*-value = 0.002) and 90 (*p*-value = 0.068), although these associations did not remain after multivariable adjustment. No significant associations were observed for the remaining landmark models (including those based on high-level viremia or recent CMV exposure), or when bacterial and opportunistic infection were separately analysed. There were no differences in viral kinetics (peak CMV viremia and area under curve of CMV viral load) either. Our findings do not support the existence of an independent association between previous CMV exposure and the overall risk of post-transplant infection, although results might be affected by power limitations.

## Graphical Abstract

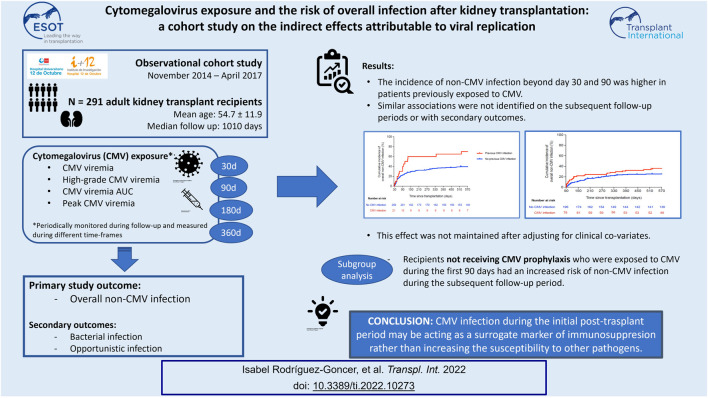

## Introduction

Despite notable advances in diagnosis, prevention and treatment, cytomegalovirus (CMV) remains as a leading cause of morbidity after solid organ transplantation (SOT) due to its direct pathogenic effects. In addition, CMV exposure is linked with a wide range of immune phenomena that would presumably exert a negative impact on the SOT population. (1, 2) These indirect effects attributable to CMV include decreased long-term graft survival (3, 4) graft rejection, (5-8) atherothrombotic events, (9, 10) new onset diabetes after transplantation (11) and a variety of bacterial and fungal infections (1, 12).

Cytomegalovirus has evolved multiple mechanisms to persist and replicate evading the host’s immune system through the impairment of antiviral responses and the enhancement of local inflammation. (13) Such immune dysfunction is known to negatively affect innate (e.g., functionality of natural killer cells and tissue macrophages) and adaptive components (e.g., cytotoxic T-cell responses). (14, 15) Besides, CMV has been shown to modulate pathways mediated by toll-like receptor ligands (16) and to promote accelerated T-cell senescence. (17, 18) These immunomodulatory effects, maintained over time, are thought to underlie the deleterious consequences allegedly caused by CMV. It is controversial, however, whether reducing CMV replication with the use of antiviral prophylaxis would impact the incidence of post-transplant events (19-22).

Virus-induced immune dysregulation may explain the association reported between CMV exposure and infections due to other microorganisms after SOT. Previous studies have suggested that CMV replication increases the risk of bacterial infection (*Listeria monocytogenes* (23, 24) or *Clostridioides difficile* (21, 25, 26)), non-CMV viral infection (hepatitis C virus (27)) and, particularly, opportunistic events such as invasive aspergillosis, (28-30) nocardiosis (31) or *Pneumocystis jirovecii* pneumonia. (22, 32, 33) It should be noted that this evidence is mainly based on retrospective case-control studies with small sample sizes. The occurrence of non-CMV infection was not the primary study outcome, and monitoring strategies used to measure CMV exposure exhibited great heterogeneity. Misclassification bias in case-control studies cannot be excluded, as recipients that had previously experienced infectious complications might have been more closely monitored for CMV replication during the subsequent follow-up. Thus, it remains unclear whether the demonstration of CMV infection merely acts as a surrogate marker for over-immunosuppression.

With these research gaps in mind, we aimed to explore the potential impact of post-transplant CMV replication on the risk of non-CMV overall infection in a large cohort of kidney transplant (KT) recipients. To overcome the aforementioned limitations, CMV exposure was assessed by means of close monitoring of CMV viremia with real-time polymerase chain reaction (PCR) and by applying various methodological strategies.

## Patients and Methods

### Study Population and Setting

We performed an observational cohort study with prospectively collected data at the University Hospital “12 de Octubre” (Madrid, Spain). All consecutive patients aged ≥18 years that underwent KT between November 2014 and April 2017 were eligible for inclusion, including double organ (e.g., kidney-pancreas and liver-kidney) recipients. Patients experiencing primary graft non-function, death or graft loss within the first week were excluded, since they had no opportunity to be exposed to CMV viremia or to experience study outcomes. The study was performed in accordance with the ethical standards laid down in the Declarations of Helsinki and Istanbul. The local Ethics Committee approved the study protocol and written informed consent was obtained from all participants at study entry.

### Study Design

All patients were enrolled at the time of transplantation and followed up until December 2018 or, alternatively, until graft loss or death. Patients were seen regularly at the outpatient transplant clinic at scheduled follow-up visits (baseline, every 2 weeks during the first 3 months, and monthly thereafter) or whenever clinically indicated. Clinical, laboratory, microbiological and histological features were prospectively collected in our institutional database by using a standardized case report form. CMV viral load was quantified by real-time PCR (as detailed below) fortnightly during the first 2 months, monthly through month 6, and every 2 months thereafter until completing the first year since transplantation, as well as at any time if clinical or laboratory manifestations suggestive of CMV disease were present.

The primary study outcome was the occurrence of non-CMV overall infection, as defined below, during the post-transplant follow-up period. Bacterial and non-CMV opportunistic infection were considered as secondary outcomes.

### Study Definitions

The diagnosis of “post-transplant infection” was established by at least one of the following criteria: 1) positive culture of an unequivocally pathogenic microorganism (e.g., *Mycobacterium tuberculosis*) from any sample; 2) isolation of any microorganism from a sample obtained under sterile conditions; 3) isolation of a potentially pathogenic microorganism from any sample accompanied by signs of local or systemic infection; and/or 4) clinical data suggestive of infection without microbiological isolation and complete resolution under antimicrobial treatment.

Febrile episodes were not taken into account if no causative agent could be demonstrated and no antimicrobial treatment was needed to achieve clinical resolution. “Pneumonia” was defined by the presence of a new infiltrate on the chest X-ray or CT scan plus one or more compatible signs or symptoms (i.e., fever or hypothermia, new cough with or without sputum production, pleuritic chest pain, dyspnea, and/or altered breath sounds on auscultation). “Lower respiratory tract infection” denoted episodes of bronchitis and/or bronchiolitis with no new pulmonary infiltrates. “Digestive tract infection” included bacterial (e.g., *Clostridioides difficile*, *Salmonella* spp. or *Campylobacter* spp.), viral (e.g., norovirus) or parasitic (helminths or protozoa) infection producing colitis and/or diarrhea. “Non-CMV viral syndrome” included episodes with typical symptoms of viral infection (e.g., fever, headache or myalgia) accompanied with compatible laboratory findings and positive microbiological identification (e.g., influenza). “Presumptive BK polyomavirus-associated nephropathy” was defined by the presence of plasma viral loads >4 log_10_ copies/ml at two time points 3 or more weeks apart. (34) Episodes of asymptomatic bacteriuria, lower urinary tract infection (i.e., cystitis) or low-level BK polyomavirus viremia were excluded.

“Non-CMV opportunistic infection” was defined as that due to intracellular bacteria (e.g., *Listeria monocytogenes, Nocardia* spp. or mycobacteria), herpesviruses (herpes simplex virus [HSV], varicella-zoster virus [VZV] and Epstein-Barr virus-related post-transplant lymphoproliferative disease), yeasts (*Candida* spp. and *Cryptococcus* spp.), molds, *P. jirovecii*, and parasites (*Cryptosporidium*, *Toxoplasma gondii* and *Leishmania* spp.). (35) “Proven or probable invasive fungal disease” was defined based on the criteria proposed by the European Organisation for Research and Treatment of Cancer and the Mycoses Study Group. (36) Bloodstream, intraabdominal, surgical site and urinary tract infections due to *Candida* spp. were excluded from the definition of opportunistic infection as these episodes are usually related to previous surgery or indwelling catheters rather than impaired immune status.

“CMV infection” was defined by the demonstration of CMV DNAemia by real-time PCR regardless of the presence of attributable symptoms or other clinical manifestations. CMV disease comprised both viral syndrome and end-organ disease. “CMV viral syndrome” was defined by the presence of CMV infection plus fever plus at least one of the following: leukopenia (white blood cell [WBC] count <3.50 × 10^3^ cells/μL if baseline WBC count was ≥4.00 × 10^3^ cells/μL or a decrease >20% if baseline WBC count was <4.00 × 10^3^ cells/μL); atypical lymphocytosis (≥5%); thrombocytopenia (platelet count <100 × 10^3^ cells/μL if baseline count was ≥115 × 10^3^ cells/μL or a decrease >20% if baseline platelet count was <115 × 10^3^ cells/μL); or elevation of ALT or AST of more than 2 times the upper limit of normal. “CMV end-organ disease” included probable or proven categories, with the latter requiring the documentation of CMV replication in tissue specimens by viral culture, immunohistochemistry, histopathology, or DNA hybridization, in the presence of attributable clinical manifestations. (37) As previously stated, CMV infection (either asymptomatic replication or clinical disease), which constituted the explanatory variable of interest, was not included in the definition of study outcomes.

The graft function was assessed by estimated glomerular filtration rate using the abbreviated Modification of Diet in Renal Disease (MDRD-4) equation. (38) “Delayed graft function” was defined as the need for dialysis within the first two post-transplant weeks. Acute graft rejection was diagnosed by histological examination if possible or by response to empirical antirejection treatment. Graft loss was defined by the permanent return to dialysis and/or retransplantation.

### Assessment of CMV Exposure

Plasma CMV DNA loads were quantified by means of a real-time PCR assay (RealStar^®^ CMV PCR kit 1.0, Altona Diagnostics GmbH, Hamburg, Germany). DNA was extracted from 200 μL of sample with the NucliSENS^®^ easyMag^®^ instrument (bioMérieux Diagnostics, Marcy l’Etoile, France), according to the manufacturer’s instructions. Viral loads were log_10_-transformed for statistical analyses. “High-level CMV viremia” was defined as a viral load ≥1,000 IU/ml. The area under curve of CMV viral load (CMV-AUC) allows for capturing viral dynamics over time by considering not only peak viral loads but also persistent replication. Therefore, we calculated CMV-AUCs (expressed as log_10_ IU × day/ml) by means of the trapezoid rule (39) from the time of transplantation to days 30 (AUC_0-30_), 90 (AUC_0-90_), 180 (AUC_0-180_) and 360 (AUC_0-360_). The CMV-AUC value for a given interval could be estimated only if at least two viral load measurements were available. We also calculated peak CMV viral loads for each of these post-transplant periods.

### Immunosuppression and Prophylaxis Regimens

Details on immunosuppressive regimens are provided in Supporting Material. All patients received preoperatively a single dose of intravenous (IV) cefazolin (or ciprofloxacin in those with ß-lactam hypersensitivity). Prophylaxis for *P. jirovecii* pneumonia was administered for 9 months with trimethoprim-sulfamethoxazole (160/800 mg three times weekly) or monthly intravenous pentamidine. In patients at high-risk for CMV infection, universal prophylaxis with oral valganciclovir (900 mg daily) was given for 3 months (seropositive recipients [R+] that received induction therapy with anti-thymocyte globulin [ATG]) or 6 months (serology mismatch [donor positive/recipient negative (D+/R−)] regardless of the type of induction therapy). Intermediate-risk patients (R+ without ATG induction) were managed by means of PCR-guided pre-emptive therapy, and IV ganciclovir (5 mg/kg/12 h) or oral valganciclovir (900 mg/12 h) for at least 2 weeks was initiated in the presence of high-level (≥1,000 IU/ml) or rapidly increasing viremia according to the criteria of the attending nephrologist. (Val)ganciclovir doses were adjusted according to renal function when necessary (1).

### Statistical Analysis

Quantitative data were shown as the mean ± standard deviation (SD) or the median with interquartile range (IQR). Qualitative variables were expressed as absolute and relative frequencies. Categorical variables were compared using the χ (2) test. Student’s *t*-test or U Mann-Whitney test were applied for continuous variables. Time-to-event curves were plotted by the Kaplan-Meier method and inter-group differences were compared with the log-rank test.

A series of landmark survival analyses were performed at days 30, 90, 180 and 360 after transplantation to evaluate the association between different approaches to CMV exposure (CMV viremia at any level, high-level CMV viremia, peak viremia and CMV-AUC) and the subsequent occurrence of non-CMV infection. Exposure to CMV was assessed within two different timeframes: throughout fixed intervals encompassing the first 30, 90, 180 and 360 days after transplantation (cumulative exposure); and through non-overlapping intervals covering the immediately preceding two-to-three-month periods (i.e., days 30–90, days 90–180, and days 270–360) (recent exposure). For each of these landmark analyses, Cox regression models were constructed with previous CMV exposure as the explanatory variable of interest and non-CMV infection as the dependant variable. Models were adjusted in a two-step process. First, a set of variables were initially tested at the univariable level. These variables encompassed demographic and clinical features of the recipient (i.e., comorbidities, causes of end-stage renal disease, previous transplantation), donor age and type (i.e., donation after brain or circulatory death, living donor), surgical and peri-operative variables (i.e., cold ischemia time, surgical complications, delayed graft function), laboratory results (i.e., graft function, leucocyte and lymphocyte count), immunosuppressive agents, occurrence of graft rejection, type of CMV prevention strategy used (antiviral prophylaxis or preemptive therapy), and the occurrence of non-CMV infection within the preceding period. Only variables achieving univariable *p*-values < 0.08 were next entered into the multivariable Cox models as potential covariates. Multicollinearity was analyzed with the variance inflation factor (VIF), with VIF values < 3 being considered acceptable. The administration of valganciclovir prophylaxis (versus preemptive therapy) was not significantly associated with the study outcome in the univariable analysis. Nevertheless, given the relevance of this variable and the potential interaction with CMV exposure we performed a set of sensitivity analyses by excluding those patients that received prophylaxis. Associations were expressed as hazard ratios (HRs) with 95% confidence intervals (CIs). Statistical analysis was performed using SPSS version 20.0 (IBM Corp., Armonk, NY) and graphs were generated with Prism version 6.0 (GraphPad Software Inc., La Jolla, CA).

## Results

### Study Population and Outcomes

Overall, 291 KT recipients were included, whose clinical characteristics are summarized in Table 1. The median follow-up was 1,010 days (IQR: 715–1,246), totalling 276,239 transplant-days. Nineteen recipients (6.5%) died at a median interval of 446 days (IQR: 38–872), accounting for 1- and 2-year survival rates of 94.8 and 93.7%, respectively. Common causes of death were infection (4 patients), malignancy and cardiovascular events (3 patients each). Twenty-two patients (7.6%) experienced graft loss, yielding 1- and 2-year death-censored graft survival rates of 94.0 and 91.8%, respectively.

**TABLE 1:** Demographics and clinical characteristics of the study population (*n* = 291).

Variable	
Age of recipient, years [mean ± SD]	54.7 ± 11.9
Gender of recipient (male) [n (%)]	201 (69.1)
Prior or current smoking history [n (%)]	111 (38.1)
BMI at transplantation, kg/m^2^ [median (IQR)]^a^	25.3 (22.3–28.4)
Pre-transplant chronic conditions [n (%)]	
Hypertension	244 (83.8)
Diabetes mellitus	88 (30.2)
Coronary heart disease	29 (10.0)
Other chronic heart disease	47 (16.2)
Peripheral arterial disease	26 (8.9)
Cerebrovascular disease	24 (8.2)
Chronic obstructive pulmonary disease	7 (2.4)
Type of transplantation [n (%)]	
Single kidney	272 (93.5)
Simultaneous kidney-pancreas	13 (4.5)
Simultaneous liver-kidney	6 (2.1)
Previous solid organ transplantation [n (%)]	36 (12.4)
Underlying cause of end-stage kidney disease [n (%)]	
Glomerulonephritis	65 (22.3)
Diabetic nephropathy	58 (19.9)
Polycystic kidney disease	39 (13.4)
Nephroangiosclerosis	23 (7.9)
Congenital nephropathy	10 (3.1)
Reflux nephropathy	8 (2.7)
Lupus nephropathy	5 (1.7)
Vasculitis	5 (1.7)
Chronic interstitial nephropathy	2 (0.7)
Unknown	32 (10.9)
Other	38 (13.1)
CMV serostatus [n (%)]	
D+/R+	208 (71.5)
D-/R+	37 (12.7)
D+/R-	31 (10.7)
D-/R-	10 (3.4)
D unknown/R+	5 (1.7)
Positive EBV serostatus (anti-EBNA IgG) [n (%)]	258 (88.7)
Positive HCV serostatus [n (%)]	25 (8.6)
Positive HBsAg status [n (%)]	10 (3.4)
Positive HIV serostatus [n (%)]	3 (1.0)
Pre-transplant renal replacement therapy [n (%)]	261 (89.7)
Hemodialysis	216 (74.2)
Continuous ambulatory peritoneal dialysis	45 (15.5)
Time on dialysis, days [median (IQR)]	572 (287.5–1,085.5)
Age of donor, years [mean ± SD]	53.2 ± 16.6
Gender of donor (male) [n (%)]	165 (56.7)
Type of donor [n (%)]	
DBD donor	185 (63.9)
DCD donor	71 (24.4)
Living donor	31 (10.7)
Cold ischemia time, hours [median (IQR)]	17 (10.3–22.3)
Number of HLA mismatches [median (IQR)]	4 (3–5)
Intraoperative blood product transfusion [n (%)]	34 (11.7)
Induction therapy [n (%)]	
ATG	146 (50.2)
Total dose, mg [mean ± SD]	4.8 ± 2.4
Basiliximab	105 (36.1)
Methylprednisolone only	40 (13.7)
Primary immunosuppression [n (%)]	
Steroids	290 (99.7)
Tacrolimus	291 (100.0)
Mycophenolate mofetil/mycophenolic acid	279 (95.9)
Azathioprine	12 (4.1)
Everolimus	1 (0.3)
CMV antiviral prophylaxis [n (%)]	166 (57.0)
Duration of prophylaxis, days [median (IQR)]	96 (90–139)
Post-transplant complications [n (%)]	
Delayed graft function	140 (48.1)
Number of dialysis sessions [median (IQR)]	2 (1–3)
Reintervention within the first month	33 (11.3)
NODAT	39 (13.4)
Renal artery stenosis requiring revascularization	23 (7.9)
Acute graft rejection^b^	40 (14.1)
>2 episodes of acute rejection	8 (2.7)
Time to the first episode, days [median (IQR]	86.5 (15–182.5)
T-cell-mediated acute rejection	21 (7.2)
Antibody-mediated acute rejection	10 (3.4)

ATG: antithymocyte globulin; BMI: body mass index; CMV: cytomegalovirus; D: donor; DBD: donation after brain death; DCD: donation after circulatory death; EBV: Epstein-Barr virus; HCV: hepatitis C virus; HBsAg: hepatitis B virus surface antigen; HIV: human immunodeficiency virus; HLA: human leukocyte antigen; IQR: interquartile range; NODAT: new-onset diabetes after transplantation; SD: standard deviation; R: recipient.

a Data on BMI, not available for 23 patients.

b Includes 7 patients with borderline acute rejection and 6 with empirically-treated episodes not confirmed by biopsy.

One-hundred and ninety-seven patients (67.7%) developed a total of 424 episodes of non-CMV infection (incidence rate of 1.54 episodes [95% CI: 1.39–1.69] per 1,000 transplant-days). Clinical syndromes and causative agents are detailed in Table 2. The median interval from transplantation to the first episode was 29.0 days (IQR: 13.0–73.5), and about one quarter of the episodes (25.2% [107/424]) occurred within the first month (mainly acute pyelonephritis, surgical site infection and other healthcare-associated infections). Regarding the secondary outcomes, 167 patients (57.4%) experienced 331 episodes of bacterial infection (incidence rate of 1.19 [95% CI: 1.07–1.33] per 1,000 transplant-days) and 34 patients (11.7%) had 41 episodes of non-CMV opportunistic infection (incidence rate of 0.15 [95% CI: 0.11–0.19] per 1,000 transplant-days), as detailed in Supplementary Table S1 in Supporting Material. Fifty-three episodes did not meet the criteria for bacterial or opportunistic infection (namely influenza [30.2%], invasive candidiasis [20.8%] and other respiratory viral infections [18.9%]).

**TABLE 2:** Clinical and microbiological description of all the episodes of non-CMV post-transplant infection occurring during the follow-up period (*n* = 424).

Clinical syndrome	N (%)
Acute graft pyelonephritis	147 (34.9)
Secondary bloodstream infection	48/147 (32.6)
Surgical site infection	46 (10.8)
Secondary bloodstream infection	4/46 (8.7)
Digestive tract infection	37 (8.7)
Secondary bloodstream infection	1/37 (2.7)
Skin and soft-tissue infection	35 (8.3)
Lower respiratory tract infection	35 (8.3)
Pneumonia	26 (6.1)
Secondary bloodstream infection	2/26 (7.7)
Viral syndrome	15 (3.5)
Intraabdominal infection	12 (2.8)
Secondary bloodstream infection	2/12 (16.7)
Catheter-related bloodstream infection	10 (2.4)
Prostatitis	4 (0.9)
CNS infection	1 (0.2)
Other	53 (12.5)
Isolated microorganisms	N (%)
Bacteria	
*Escherichia coli*	87 (20.5)
*Klebsiella pneumoniae*	59 (13.9)
*Pseudomonas aeruginosa*	37 (8.7)
*Clostridioides difficile*	20 (4.7)
*Enterococcus faecalis*	18 (4.2)
*Enterococcus faecium*	16 (3.8)
Other *Enterobacteriaceae*	11 (2.6)
Coagulase-negative staphylococci	7 (1.7)
*Staphylococcus aureus*	6 (1.4)
*Enterobacter* spp.	4 (0.9)
*Campylobacter* spp.	4 (0.9)
*Streptococcus pneumoniae*	2 (0.5)
*Serratia marcescens*	2 (0.5)
Non-typhoidal *Salmonella*	1 (0.2)
*Nocardia* spp.	1 (0.2)
Other	15 (3.5)
No microbiological diagnosis^a^	39 (9.2)
Viruses	
Influenza virus	16 (3.8)
HSV-1/2	13 (3.0)
Varicella-zoster virus	12 (2.8)
Respiratory syncytial virus	6 (1.4)
BK polyomavirus^b^	4 (0.9)
Human metapneumovirus	2 (0.5)
Norovirus	1 (0.2)
Erythrovirus B19	1 (0.2)
Other	13 (3.0)
Fungi	
*Candida* spp.	16 (3.8)
*Aspergillus* spp.	4 (0.9)
Mucorales	2 (0.5)
*Cryptococcus neoformans*	1 (0.2)
*Pneumocystis jirovecii*	1 (0.2)
Parasites	
*Strongyloides stercoralis*	1 (0.2)
*Giardia lamblia*	1 (0.2)

CNS: central nervous system; HSV: herpes simplex virus.

a The presumptive diagnosis of bacterial infection was established by the complete clinical resolution with antibiotic therapy in the absence of an alternative cause.

b Presumptive BK, polyomavirus-associated nephropathy (i.e., plasma viral load >4 log^10^ copies/ml at two time points three or more weeks apart).

### CMV Exposure

One hundred and sixty-six patients (57.0%) received antiviral prophylaxis with valganciclovir for a median of 96 days (IQR: 90–139), whereas the remaining of the cohort was managed with pre-emptive therapy. The total number of monitoring points for CMV DNAemia throughout the entire follow-up period was 3,177, with a median of 11 points per patient (IQR: 8–13).

Incidence, clinical characteristics and viral kinetics of CMV events are shown in Table 3. Overall, 146 patients (50.2%) experienced at least one episode of CMV infection, either as asymptomatic viremia (78.1% [114/146]) or clinical disease (21.9% [32/146]). About one third of the patients with asymptomatic CMV infection (34.2% [39/114]) actually received pre-emptive antiviral therapy with (val)ganciclovir at any time. The median load in episodes of viremia requiring or not requiring pre-emptive therapy was 3.5 log_10_ IU/ml (IQR: 3.2–3.9) and 2.9 log_10_ IU/ml (IQR: 2.5–3.6), respectively.

**TABLE 3:** Incidence, clinical characteristics and viral kinetics parameters of CMV events.

Asymptomatic CMV infection	
Number of patients with at least one episode	114
Cumulative incidence, % (95% CI)	39.2 (33.5–45.0)
Interval from transplantation to the first episode, days [median (IQR)]	71.0 (35.8–149.3)
Late-onset infection (beyond day 180), n (%)^a^	53/269 (19.7)
Requirement for pre-emptive therapy, n (%)	39/114 (34.2)
Patients with recurrent infection, n (%)^b^	27 (23.7)
Number of episodes of viremia	166
Peak viral load, log_10_ IU/ml [median (IQR)]	3.2 (2.7–3.8)
Episodes requiring antiviral therapy	42/166 (25.3)
Viral load, log_10_ IU/ml [median (IQR)]	3.5 (3.2–3.9)
Episodes not requiring antiviral therapy	124/166 (74.7)
Viral load, log_10_ IU/ml [median (IQR)]	2.9 (2.5–3.6)
CMV-AUC_0-360_, log_10_ IU × days × ml^−1^ [median (IQR)]	4.7 (4.1–5.2)
CMV disease	
Number of patients with at least one episode	32
Cumulative incidence, % (95% CI)	11.0 (7.4–14.6)
Interval from transplantation to the first episode, days [median (IQR)]	50.0 (34.0–176.5)
Clinical syndrome [n (%)]	
Viral syndrome	27/32 (84.4)
Colitis	4/32 (12.5)
Hepatitis	1/32 (3.1)

CI: interval confidence; CMV: cytomegalovirus; CMV-AUC: area under curve of CMV viral load; IQR: interquartile range.

a Percentage calculated on the basis of those KT, recipients that remained alive with a functioning graft by day 180 after transplantation (*n* = 269).

b At least two episodes separated by both a minimum 2-weeks interval and at least one negative sample for CMV DNA.

### Association Between Cumulative CMV Exposure and Overall Non-CMV Infection

**FIGURE 1:**
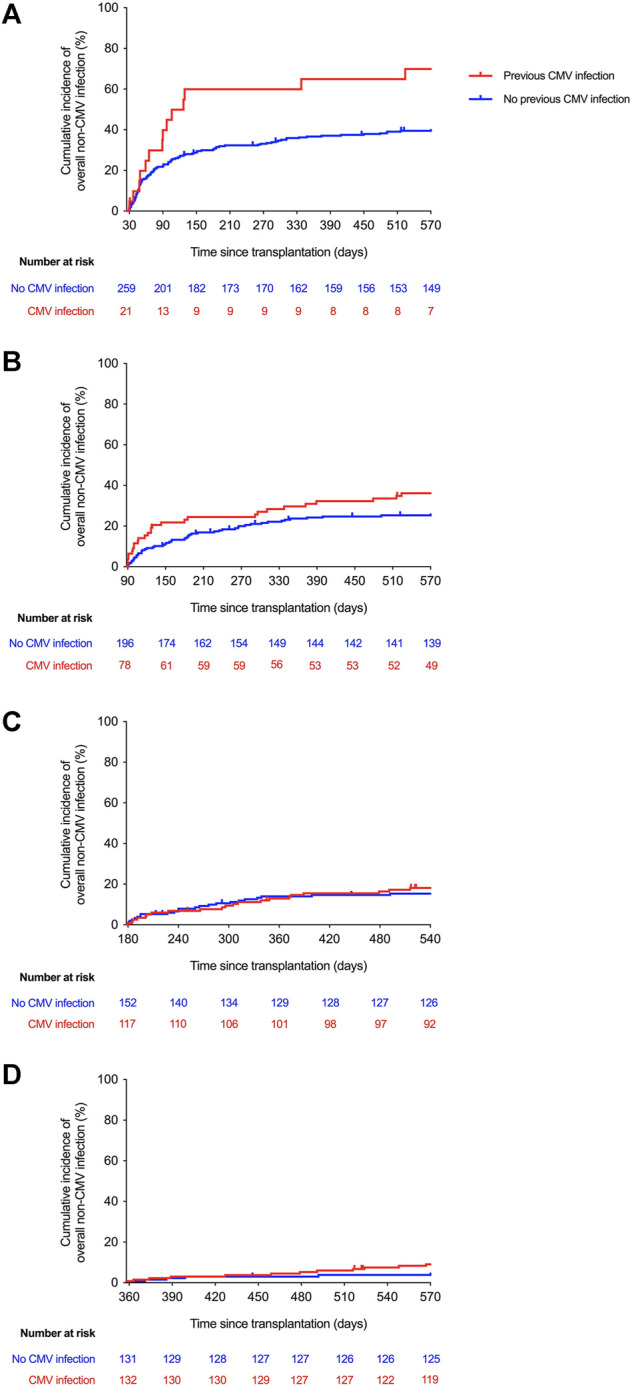
Kaplan-Meier curves for the incidence of overall non-CMV infection according to the cumulative exposure to CMV infection at any level beyond day 30 (log-rank *p*-value = 0.002) **(A)**, day 90 (log-rank *p*-value = 0.068) **(B)**, day 180 (log-rank *p*-value = 0.727) **(C)**, and day 360 (log-rank *p*-value = 0.314) **(D)** after transplantation. CMV: cytomegalovirus.

First, we explored the association between CMV infection throughout fixed intervals after transplantation (i.e., cumulative CMV exposure) and the subsequent development of non-CMV infection. The incidence of non-CMV infection beyond day 30 was significantly higher for patients with previous exposure to CMV infection at any level compared to those that had remained free from this event until day 30 (2-year incidence rates: 69.9 versus 40.8%, respectively; log-rank *p*-value = 0.002; crude HR: 2.27; 95% CI: 1.32–3.91; *p*-value = 0.003), whereas a near significant difference was observed beyond day 90 (2-year incidence rates: 36.1 versus 26.5%; log-rank *p*-value = 0.068; crude HR: 1.51; 95% CI: 0.97–2.36; *p*-value = 0.070). There were no significant differences at days 180 or 360 after transplantation (Figure 1). Similar trends were found when only high-level CMV viremia was considered, although none of the differences achieved statistical significance (Supplementary Figure S1).

**FIGURE 2:**
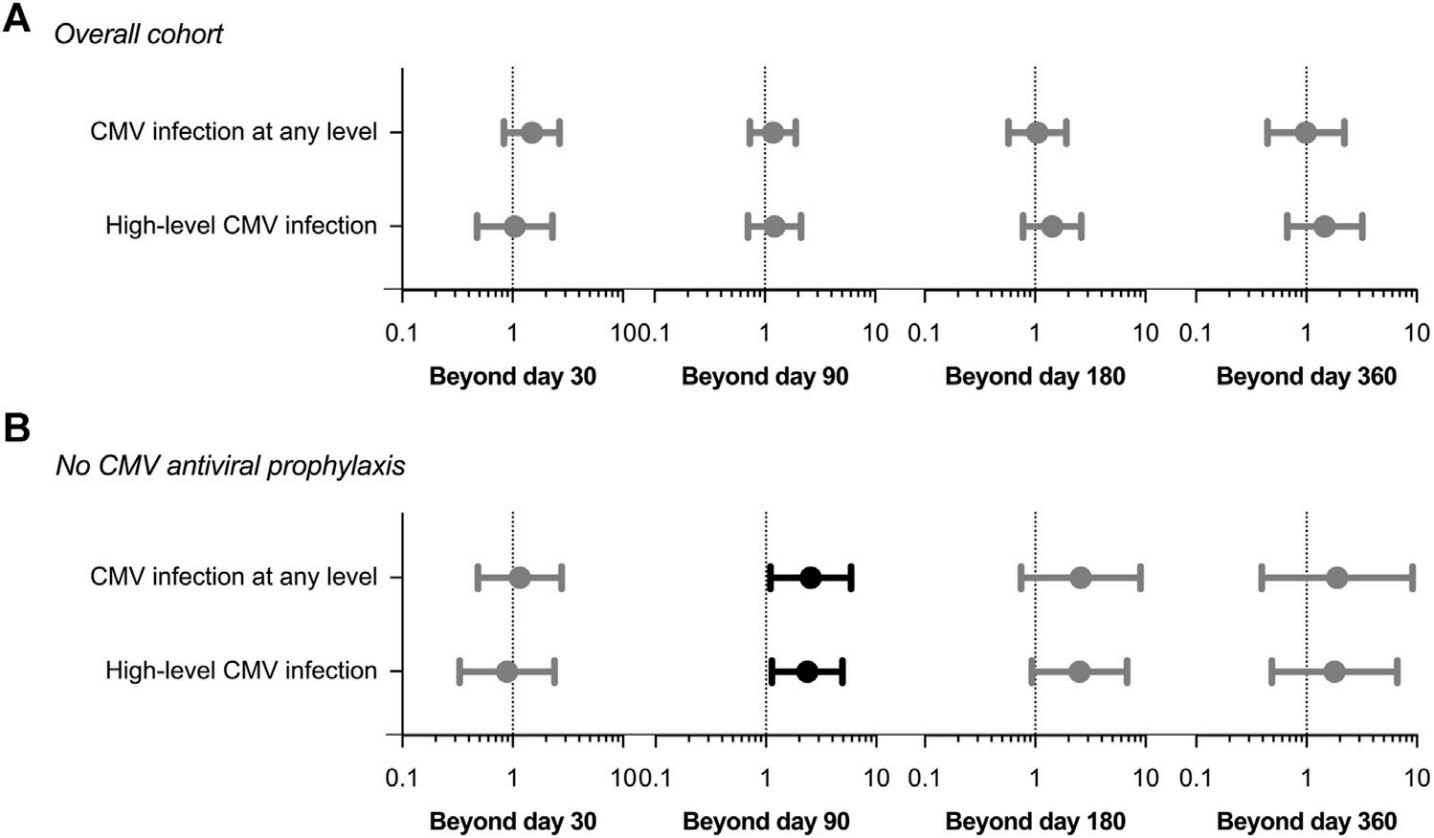
Adjusted hazard ratios (circles) with 95% confidence intervals (whiskers) in landmark Cox regression models for the occurrence of overall non-CMV infection according to the previous development of CMV infection at any level or high-level CMV infection (>1,000 IU/ml): **(A)** entire study cohort; **(B)** patients not receiving CMV antiviral prophylaxis. Clinical covariates adjusted for are listed in Supplementary Table S2. CMV: cytomegalovirus.

After adjusting for those covariates that have been previously proven to achieve univariable *p*-values < 0.08 (listed in Supplementary Table S2) the exposure to CMV infection during the first 30 days was no longer associated with the occurrence of non-CMV infection beyond that point (adjusted HR: 1.45; 95% CI: 0.84–2.68; *p*-value = 0.172). No significant associations were found for the remaining landmark Cox models either (Figure 2A and Supplementary Table S3).

In the sensitivity analysis restricted to the subgroup of patients that did not receive CMV antiviral prophylaxis (*n* = 125), the development during the first 90 days of CMV infection at any level (adjusted HR: 2.54; 95% CI: 1.09–5.90; *p*-value = 0.030) or high-level viremia (adjusted HR: 2.37; 95% CI: 1.14–4.95; *p*-value = 0.021) was associated with subsequent non-CMV infection, with borderline significance. Similar associations were not observed for the remaining landmark analyses (Figure 2B and Supplementary Table S3).

### Association Between Recent CMV Exposure and Overall Non-CMV Infection

Next, we exclusively considered CMV infection that occurred through non-overlapping 2- to 3-months intervals (days 30–90, days 90–180, and days 270–360) prior to the corresponding landmark time point (i.e., recent CMV exposure). There were no differences in the incidence of non-CMV infection beyond days 90, 180 or 360 between patients experiencing or not experiencing CMV infection at any level during the preceding period (Supplementary Figure S2). Accordingly, no significant associations were found in any of the adjusted Cox models (Supplementary Figure S3A). In the sensitivity analysis restricted to patients not receiving antiviral prophylaxis, however, recent high-level CMV exposure was associated (although with borderline significance) with the occurrence of non-CMV infection beyond day 90 (adjusted HR: 2.28; 95% CI: 1.06–4.70; *p*-value = 0.036) (Supplementary Figure S3B).

### Kinetics of CMV Replication and Overall Non-CMV Infection

**FIGURE 3:**
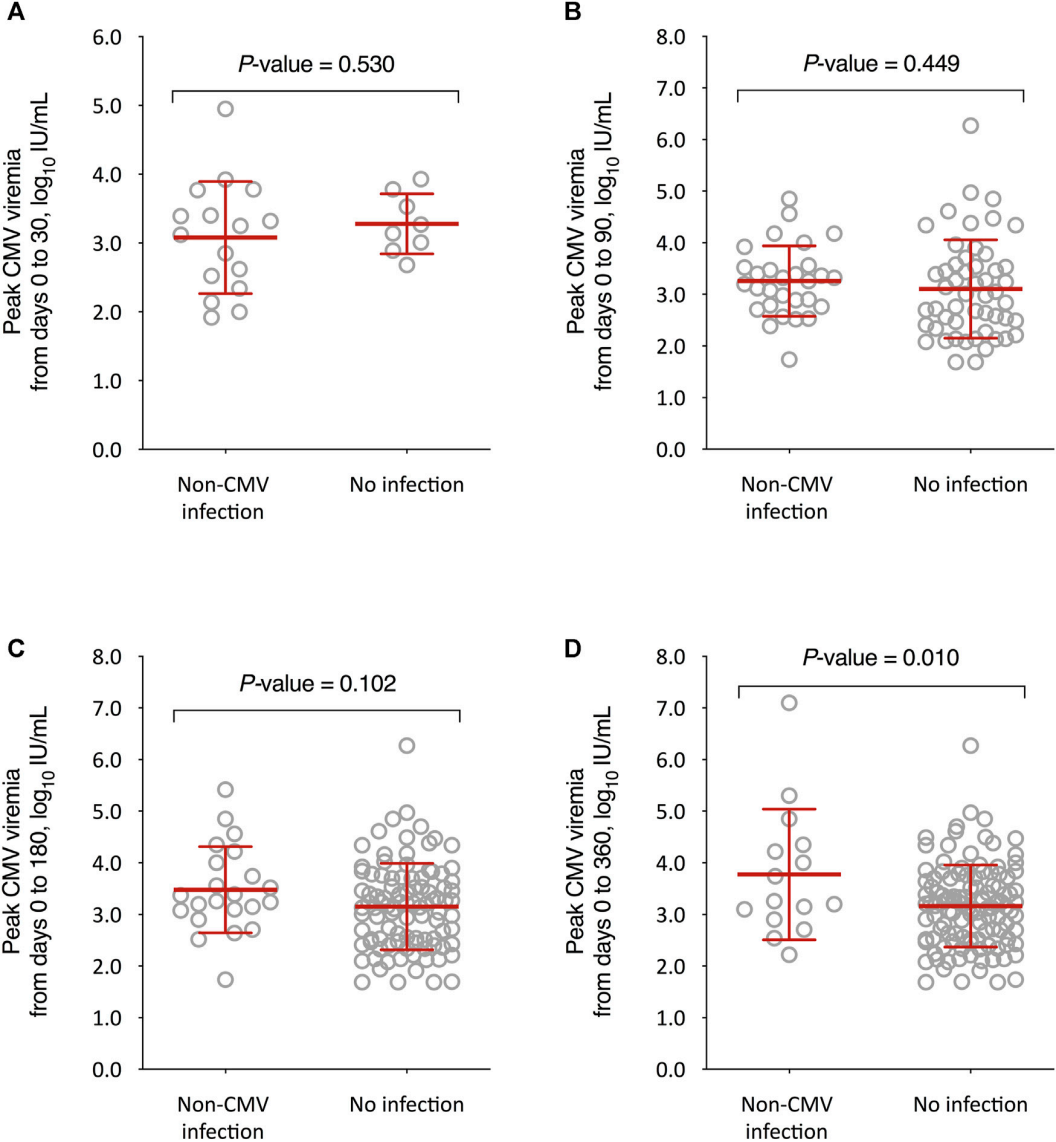
Comparison of peak CMV viral loads between KT recipients developing or not developing overall non-CMV infection beyond day 30 **(A)**, day 90 **(B)**, day 180 **(C)**, and day 360 **(D)** after transplantation. Student’s *t*-test for unpaired data was used. Bars represent mean values and whiskers the standard deviations. CMV: cytomegalovirus.

**FIGURE 4:**
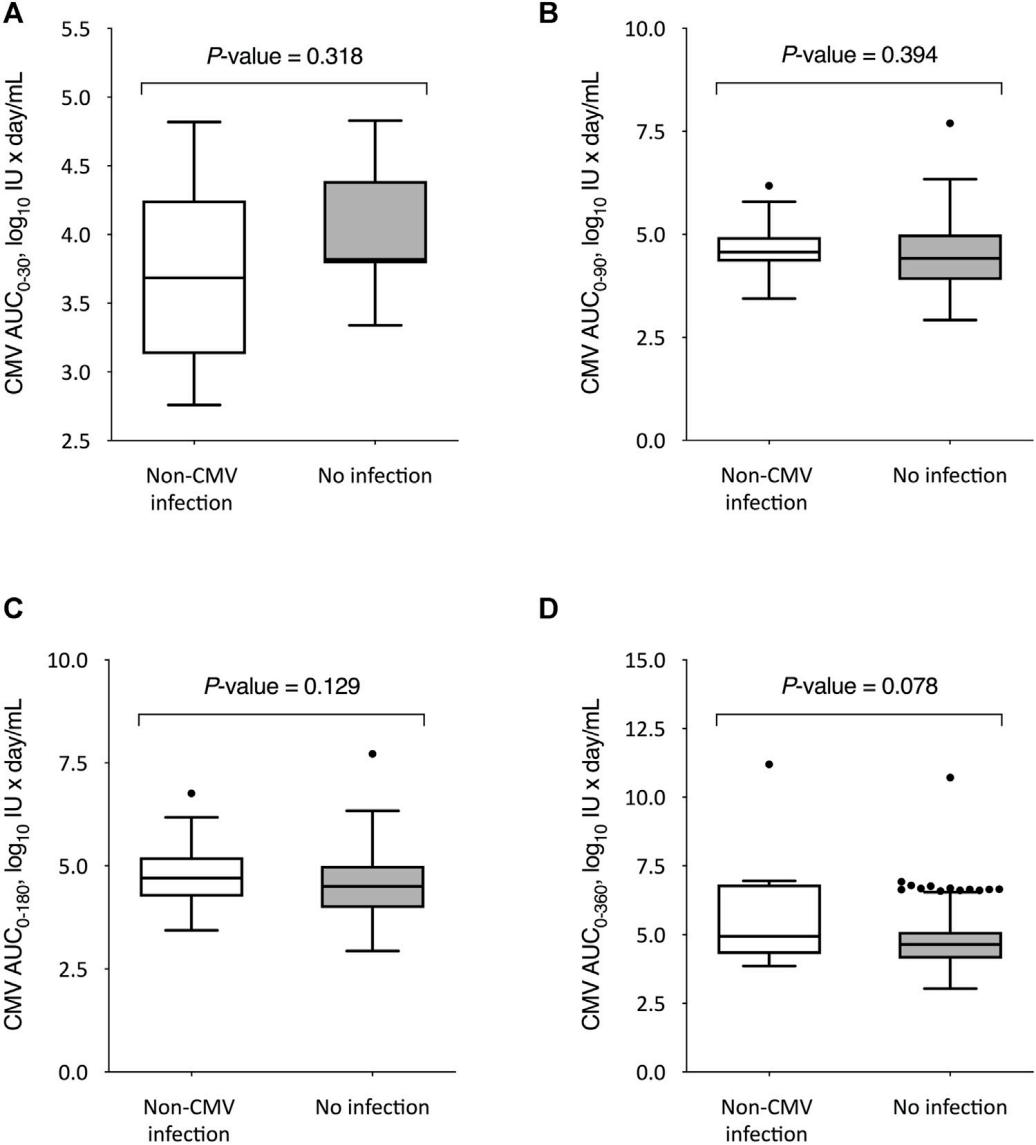
Comparison of CMV-AUCs between KT recipients developing or not developing overall non-CMV infection beyond day 30 **(A)**, day 90 **(B)**, day 180 **(C)**, and day 360 **(D)** after transplantation. Student’s *t*-test for unpaired data was used for all comparisons except for day 360, for which the U Mann-Whitney test was applied. The boxes present the interquartile distances, the horizontal lines the median, the whiskers the maximum and minimum values within 1.5 times the interquartile range, and the points the outliers. CMV-AUC: area under curve of cytomegalovirus viral load.

We also compared the kinetics of CMV DNAemia according to the subsequent occurrence of non-CMV infection. The peak CMV viral load through day 360 after transplantation was significantly higher among recipients that experienced non-CMV infection beyond that point as compared to those without this event (3.8 ± 1.3 versus 3.2 ± 0.8 log_10_ IU/ml, respectively; *p*-value = 0.010), with no differences for the remaining intervals (Figure 3). On the other hand, no significant differences were observed in the CMV-AUCs assessed through the first 30, 90, 180 and 360 days after transplantation (Figure 4).

### Secondary Outcomes

There were no significant differences in the incidence of bacterial infection at the different landmark time points according to the cumulative exposure to CMV infection at any level (Supplementary Figure S4) or high-level CMV viremia (data not shown). Likewise, no differences were observed for non-CMV opportunistic infection either (Supplementary Figure S5).

## Discussion

Several reports have suggested that CMV would increase the risk of certain non-CMV infections in SOT and allo-HSCT recipients, (12, 22, 23, 25, 27, 29, 32, 33, 40, 41) which provided the clinical foundation for hypothesizing about its presumptive immunomodulatory effects. Nevertheless, these previous studies suffer from a number of methodological flaws, including the heterogeneous—and often imprecise—approaches to define the main explanatory variable (CMV exposure), such as donor or recipient serostatus, (12,30,42) viral culture, (28) pp65 antigenemia (22) or a combination of these approaches. (23, 29) Some studies only considered CMV clinical disease but not asymptomatic infection, (25, 31, 43) or were focused on specific opportunistic agents rather than capturing the entire spectrum of non-CMV infections. (22-24, 31-33) On the other hand, most of them did not attempt to explore the potential biological gradient between the amount and length of exposure to CMV and the incidence of non-CMV infection (28, 29, 32, 33, 40).

In the present single-center cohort study comprising 291 consecutive KT recipients, CMV replication was assessed by real-time PCR to investigate in a real-life scenario the potential impact of CMV exposure on the development of non-CMV infection. We applied a variety of methodological approaches (fixed intervals versus two-to three-month periods immediately prior to the landmark time point) to take into account not only the cumulative but also the recent CMV replication, and compared different viral parameters (any level and high-level viremia, peak CMV viremia and CMV-AUC) to capture changing replication kinetics. To align our work with prior research in the field and to allow for result comparison across studies, we used a rather inclusive definition for the primary outcome (i.e., “non-CMV overall infection” due to any potentially pathogenic microorganism). In addition, we separately analysed bacterial and opportunistic infection as secondary outcomes. In doing so we attempted to dissect the potential association between CMV replication and various forms of infection in whose pathogenesis different immune arms—innate, humoral and cellular—are involved. Therefore, our approach was relatively “hypothesis-free” regarding the specific type of infection to which CMV could eventually be contributing.

The most notable finding of our study was that KT recipients early exposed to CMV replication at any level during the first 30 days after transplantation exhibited a higher incidence of non-CMV infection over the following months. A similar trend, although non-significant, was also observed beyond post-transplant day 90. After adjusting for clinically relevant covariates, such as recipient age, comorbidities, type of donor, need of reintervention or graft function (as detailed in Supplementary Table S2), however, this effect was not sustained. Moreover, no significant associations were observed between cumulative CMV exposure and the subsequent occurrence of infection beyond days 180 or 360, or with bacterial and non-CMV opportunistic infections separately considered. We initiated landmark analyses by day 30, considering that most infections occurring earlier after transplantation were hospital-acquired (i.e., surgical site or catheter-related) and therefore hardly attributable to CMV, and that CMV infection typically occurs only after the first month. No apparent impact was observed when the causal relationship was temporally delineated in terms of recent CMV exposure either, by considering only the episodes of CMV infection that occurred in the preceding period. Finally, no evidence of dose-response gradient between the amount of CMV exposure—measured as high-level viremia, peak viral load or CMV-AUC—and the occurrence of non-CMV infection was found.

The results of the present study overall suggest that CMV replication would act as a surrogate marker of immunosuppression during the initial post-transplant period (first 30 and likely 90 days) rather than actually playing a causative role in the susceptibility to other pathogens. If CMV infection constitutes an independent risk factor for non-CMV infection, it would have been expected that this cause-and-effect relationship would have been evident throughout the entire follow-up and in particular beyond day 180, when drug-induced immunosuppression is usually reduced. No impact was observed for the specific outcome of opportunistic infection either, also supporting the notion that the immunomodulatory mechanisms deployed by CMV *in vitro* have no clinically meaningful effects. Nevertheless, the lack of apparent association for the late post-transplant period might be at least partially explained by the fact that most episodes of CMV viremia occurred during the first months after transplantation (with only 19.5% of patients experiencing late-onset infection), which could have contributed to dilute the potential effect (if any). On the other hand, it is unclear how long the alleged immunomodulatory actions resulting from active or recent CMV infection would last, although our results would be consistent with some type of effect at least early after KT.

The clinical impact of CMV antiviral prophylaxis on the risk of non-CMV infection after SOT or allo-HSCT remains controversial. A meta-analysis that compiled the results of 17 trials found a lower incidence of bacterial or fungal infections among patients that received prophylaxis, whereas no statistically significant reduction was observed with the pre-emptive approach. (44) Nevertheless, an updated meta-analysis performed by the same authors that included both direct and indirect comparisons across 20 studies reported no differences in the incidence of HSV, VZV, bacterial or fungal infection according to the strategy used. (45) A randomized clinical trial that compared 6-months valganciclovir versus PCR-guided CMV pre-emptive therapy in allo-HSCT recipients found no differences in the rates of bacterial or fungal infection between both arms. (46) In the same line, no apparent advantages of valganciclovir prophylaxis in terms of non-CMV infection were observed in a recently published trial in high-risk (D+/R−) liver transplant recipients. (47) One key finding of the present study was that, after multivariable adjustment, the development of any level or high-level CMV viremia through day 90 was marginally associated with subsequent non-CMV infection in the sensitivity analysis restricted to KT recipients that did not receive antiviral prophylaxis. A similar result was observed when only recent CMV exposure (from days 30 to 90) was considered. Again, such an association was not reproduced for the remaining periods, which supports the role of CMV replication as a marker of immunosuppression early after transplantation. Nevertheless, this subanalysis should be taken with caution due to the lower number of patients included. It should be noted that the frequency of monitoring points for CMV DNAemia in the group under prophylaxis was close to that of pre-emptively managed patients (median of 10 and 12 monitoring points, respectively). Interestingly, the use of antiviral prophylaxis per se (versus preemptive therapy) exerted no direct impact on the risk of non-CMV infection regardless of the landmark time point considered (data not shown). Thus, it is plausible that the presumed role of CMV infection as a surrogate marker of immunosuppression would only operate in those recipients managed by preemptive therapy, since valganciclovir prophylaxis effectively abrogates viral replication even in the most severely immunocompromised patients. An alternative explanation resides in the fact that the main indication for antiviral prophylaxis was previous induction therapy with ATG, leading to a potential interaction between both variables. Indeed, CMV exposure would add little in recipients already experiencing ATG-induced long-term T-cell depletion. This modification of effect, on the contrary, would not be present in the group of preemptive therapy.

Our research has some limitations to be acknowledged. Firstly, this is a single-centre study and the differential impact of local monitoring practices cannot be excluded. Since half of the patients in our cohort received ATG as induction therapy, immunosuppression and prophylaxis regimens may not be applicable to other institutions. Due to the observational design, CMV monitoring was performed as usual clinical practice and may have not been as stringent as recommended in guidelines. (1) The number of episodes of non-CMV opportunistic infection—mainly due to HSV and VZV—was low, limiting the statistical power of this secondary analysis. Immunosuppressive drug levels at different time points were not available. Caution should be exercised when interpreting the comparison between CMV-AUCs, as the administration of antiviral therapy among preemptively managed patients alters viral kinetics. Finally, it should be noted that most of the non-CMV infection episodes occurred during the first 3–6 post-transplant months (median of 29.0 days to the first episode). Therefore, it is not possible to completely rule out a biologically relevant effect of CMV replication in later periods due to low statistical power.

On the other hand, the present study comprised a large and well-characterized cohort of consecutive KT recipients with a prolonged follow-up, allowing us to detect the occurrence of late events. In addition, the close monitoring of CMV viremia by molecular methods and the combination of different methods to measure viral dynamics strengthen our results. By performing different landmark survival analysis with separate Cox models for each period considered, we were able to test the potential impact of cumulative exposure to CMV across various post-transplant periods with changing immunosuppression load, (48) although alternative approaches—for instance, treating the variable of “CMV exposure” as a time-varying covariate in a single Cox model—would have been also reasonable.

In conclusion, CMV exposure (cumulative, recent or at high level) was not independently associated with an apparent increase in the subsequent risk of non-CMV infection in this cohort of KT recipients. Taken together, our findings do not clearly support the hypothesis that the immunomodulatory effects driven by CMV replication exert an impact on the overall risk of infection and would rather point to its role as a surrogate marker of immunosuppression, particularly during the first months after transplantation and in KT recipients under preemptive therapy. Further studies are needed to unravel the complex interplay between CMV and the susceptibility to post-transplant infection.

## Data Availability

The raw data supporting the conclusions of this article will be made available by the authors, without undue reservation.

## Abbreviations

allo-HSCT, allogeneic stem-cell transplantation; ATG, anti-thymocyte globulin; CI, confidence interval; CMV, cytomegalovirus; CMV-AUC, area under curve of CMV viral load; D, donor; HR, hazard ratio; HSV, herpes simplex virus; IQR, interquartile range; IV, intravenous; KT, kidney transplantation; PCR, polymerase chain reaction; R, recipient; SD, standard deviation; SOT, solid organ transplantation; VIF, variance inflation factor; VZV, varicella-zoster virus; WBC, white blood cell.
