# Supplementary material for: Cytomegalovirus Exposure and the Risk of Overall Infection After Kidney Transplantation: A Cohort Study on the Indirect Effects Attributable to Viral Replication
Source: Transpl Int. 2022 Jan 20;35:10273. doi: 10.3389/ti.2021.10273 (PMC8842254; doi:10.3389/ti.2021.10273)

Supporting information

Supporting Methods

Description of immunosuppressive regimen

All recipients of organs from donors after circulatory death underwent induction therapy with rabbit anti-thymocyte globulin (ATG-Fresenius, 1.25 mg/Kg daily for 5-7 days), with delayed introduction of a calcineurin inhibitor (CNI) from post-transplant day 6. Recipients at high immunological risk also received ATG induction for 1-3 days with early CNI initiation from post-transplant day 0. Basiliximab (20 mg on days 0 and 4) with delayed CNI introduction from post-transplant day 5 was reserved to patients at high risk for CNI-related nephrotoxicity (i.e., advanced age or pre-transplant comorbidities). Maintenance immunosuppression regimen consisted of tacrolimus (0.1 mg/Kg daily, adjusted to a target trough level of 10-15 ng/mL during the first month and 5-10 ng/mL thereafter); mycophenolic acid (360 mg twice daily); and prednisone (1 mg/Kg daily with progressive tapering). Conversion to mammalian target of rapamycin (mTOR) inhibitor-based regimens with reduced-dose tacrolimus (target trough level of 3-6 ng/mL) was performed on an individual basis for recipients experiencing severe CNI-related adverse effects, difficult-to-treat cytomegalovirus infection, or post-transplant malignancy.

Supporting Results

Table S1. Description of episodes of non-CMV opportunistic infection (n = 41).

Clinical syndrome	N (%)
Mucocutaneous HSV-1/2 infection	13 (31.7)
Herpes zoster (shingles)	12 (29.3)
Probable invasive pulmonary aspergillosis	4 (9.8)
Mucosal and cutaneous candidiasis	3 (7.3)
Proven mucormycosis	2 (4.9)
Candida esophagitis	2 (4.9)
Cryptococcal meningitis	1 (2.4)
Invasive trichosporonosis	1 (2.4)
Pneumocystis jiroveci pneumonia	1 (2.4)
Nocardiosis	1 (2.4)
Visceral leishmaniasis	1 (2.4)

HSV: herpes simplex virus.

Table S2. Clinical variables not related to CMV exposure that achieved univariable *P*-values <0.08 and were entered as covariates in landmark Cox regression models[a].

Non-CMV infection beyond day 30 after transplantation				
	**No infection** (n = 159)	**Infection** (n = 122)	**Univariable HR (95% CI)**	***P*-value**
Age of recipient, years [mean ± SD]	52.5 ± 15.8	57.0 ± 15.5	1.02 (1.00 – 1.03)[b]	0.011
Number of pre-transplant comorbidities [median (IQR)]	2 (1 – 3)	2 (1 – 4)	1.13 (1.00 – 1.27)[b]	0.043
Pre-transplant cerebrovascular disease [n (%)]	8 (5.1)	16 (13.1)	1.89 (1.12 – 3.19)	0.018
Age of the donor, years [men ± SD]	50.9 ± 16.2	55.3 ± 16.6	1.01 (1.00 – 1.02)[b]	0.017
Living donor [n (%)]	23 (14.6)	8 (6.6)	0.47 (0.23 – 0.96)	0.039
Intraoperative blood product transfusion [n (%)]	13 (8.2)	19 (15.6)	1.59 (0.98 -2.59)	0.063
Delayed graft function [n (%)]	67 (42.1)	66 (54.5)	1.53 (1.07 – 2.19)	0.020
Reintervention within the first month [n (%)]	11 (6.9)	18 (14.8)	2.03 (1.23 – 3.35)	0.006
Estimated GFR at month 1, mL/min/1.72 m^2 [mean ± SD]	44.0 ± 19.6	36.1 ± 17.8	0.98 (0.97 – 0.99)[b]	<0.001
Non-CMV infection within the first month [n (%)]	39 (24.5)	52 (42.6)	2.01 (1.40 – 2.88)	<0.001
Non-CMV infection beyond day 90 after transplantation				
	**No infection** (n = 190)	**Infection** (n = 84)	**Univariable HR (95% CI)**	***P*-value**
Number of HLA mismatches [median (IQR)]	4 (3 – 5)	5 (3.3 – 5)	1.17 (0.99 – 1.38)[b]	0.067
CMV serostatus D+/R- [n (%)]	16 (8.4)	13 (15.5)	1.72 (0.95 – 3.10)	0.074
Positive HCV serostatus [n (%)]	21 (11.4)	3 (3.6)	2.83 (0.89 – 8.96)	0.077
Reintervention within the first 3 months [n (%)]	18 (9.5)	15 (17.9)	1.75 (1.00 – 3.07)	0.049

Estimated GFR at month 3, mL/min/1.72 m^2 [mean ± SD]	45.6 ± 16.3	40.6 ± 17.9	0.98 (0.97 – 0.99)[b]	0.009
Non-CMV infection within the first 3 months [n (%)]	73 (38.4)	54 (64.3)	2.49 (1.59 – 3.89)	<0.001
Acute graft rejection within the first 3 months [n (%)]	9 (4.7)	8 (9.5)	1.98 (0.96 – 4.10)	0.066

Non-CMV infection beyond day 180 after transplantation

	**No infection** (n = 214)	**Infection** (n = 52)	**Univariable HR (95% CI)**	***P*-value**
Pre-transplant cerebrovascular disease [n (%)]	14 (6.4)	10 (19.2)	2.82 (1.41 – 5.63)	0.003
Number of HLA mismatches [median (IQR)]	4 (3 – 5)	5 (3 – 6)	1.22 (0.98 – 1.52)[b]	0.074
CMV serostatus D+/R- [n (%)]	20 (9.1)	9 (17.3)	2.06 (1.00 – 4.22)	0.050
Non-CMV infection within the first 6 months [n (%)]	104 (47.5)	35 (67.3)	2.19 (1.23 – 3.91)	0.008

Non-CMV infection beyond day 360 after transplantation

	**No infection** (n = 232)	**Infection** (n = 32)	**Univariable HR (95% CI)**	***P*-value**
Age of the donor, years [men ± SD]	51.6 ± 16.5	57.6 ± 17.9	1.02 (0.99 – 1.04)[b]	0.059
Pre-transplant cerebrovascular disease [n (%)]	14 (6.1)	8 (25.0)	4.20 (1.89 – 9.37)	0.0004
Non-CMV infection within the first 12 months [n (%)]	119 (51.3)	26 (81.2)	3.78 (1.56 – 9.19)	0.003

CI: confidence interval; CMV: cytomegalovirus; D: donor; HCV: hepatitis C virus; GFR: glomerular filtration rate; HLA: human leukocyte antigen; HR: hazard ratio; IQR: interquartile range; SD: standard deviation; R: recipient.

[a] Various groups of variables were initially tested at the univariable level: demographic and clinical features (i.e. comorbidities, causes of end-stage renal disease, previous transplantation), donor age and type (i.e. donation after brain or circulatory death, living donor), surgical and peri-operative variables (i.e. cold ischemia time, surgical complications, delayed graft function), laboratory results (i.e. graft function, leucocyte and lymphocyte count), immunosuppressive agents, occurrence of graft rejection, type of CMV prevention strategy used (antiviral prophylaxis or preemptive therapy), and the occurrence of non-CMV infection within the preceding period. Only those variables that achieved univariate *P*-values <0.08 were entered into the different Cox models, providing that the amount of collinearity was acceptable.

[b] HR per unitary increment.

Table S3. Multivariable Cox regression models for different landmark survival analyses analyzing the impact of the cumulative exposure to any level or high-level (≥1,000 IU/mL) CMV viremia on the subsequent occurrence of overall non-CMV infection, both in the entire cohort and in the subgroup of patients that did not receive CMV antiviral prophylaxis.

Landmark survival analysis		**Overall cohort** (n = 291)			**No antiviral prophylaxis** (n = 125)		
		aHR	95% CI	*P*-value	aHR	95% CI	*P*-value[a]
Beyond day 30	CMV infection at any level	1.499	0.839 – 2.679	0.267	1.163	0.486 – 2.786	0.342
	High-level CMV viremia	1.045	0.476 – 2.294	0.456	0.891	0.331 – 2.398	0.456
Beyond day 90	CMV infection at any level	1.184	0.732 – 1.915	0.567	2.542	1.095 – 5.901	**0.030**
	High-level CMV viremia	1.226	0.705 – 2.132	0.898	2.371	1.136 – 4.947	**0.021**
Beyond day 180	CMV infection at any level	1.050	0.574 – 1.923	0.345	2.588	0.744 – 9.005	0.385
	High-level CMV viremia	1.434	0.780 – 2.638	0.390	2.518	0.931 – 6.812	0.256
Beyond day 360	CMV infection at any level	0.990	0.443 – 2.212	0.401	1.895	0.394 – 9.118	0.454
	High-level CMV viremia	1.468	0.671 – 3.212	0.560	1.799	0.486 – 6.660	0.348

aHR: adjusted hazar ratio; CI: confidence interval; CMV: cytomegalovirus.

[a] Bold characters denote significant associations.

Figure S1. Kaplan-Meier curves for the incidence of overall non-CMV infection (primary outcome) according to the cumulative exposure to high-level CMV viremia (≥1,000 IU/mL) beyond day 30 (log-rank *P*-value = 0.094) **(a)**, day 90 (log-rank *P*-value = 0.086) **(b)**, day 180 (log-rank *P*-value = 0.208) **(c)**, and day 360 (log-rank *P*-value = 0.137) **(d)** after transplantation. CMV: cytomegalovirus.

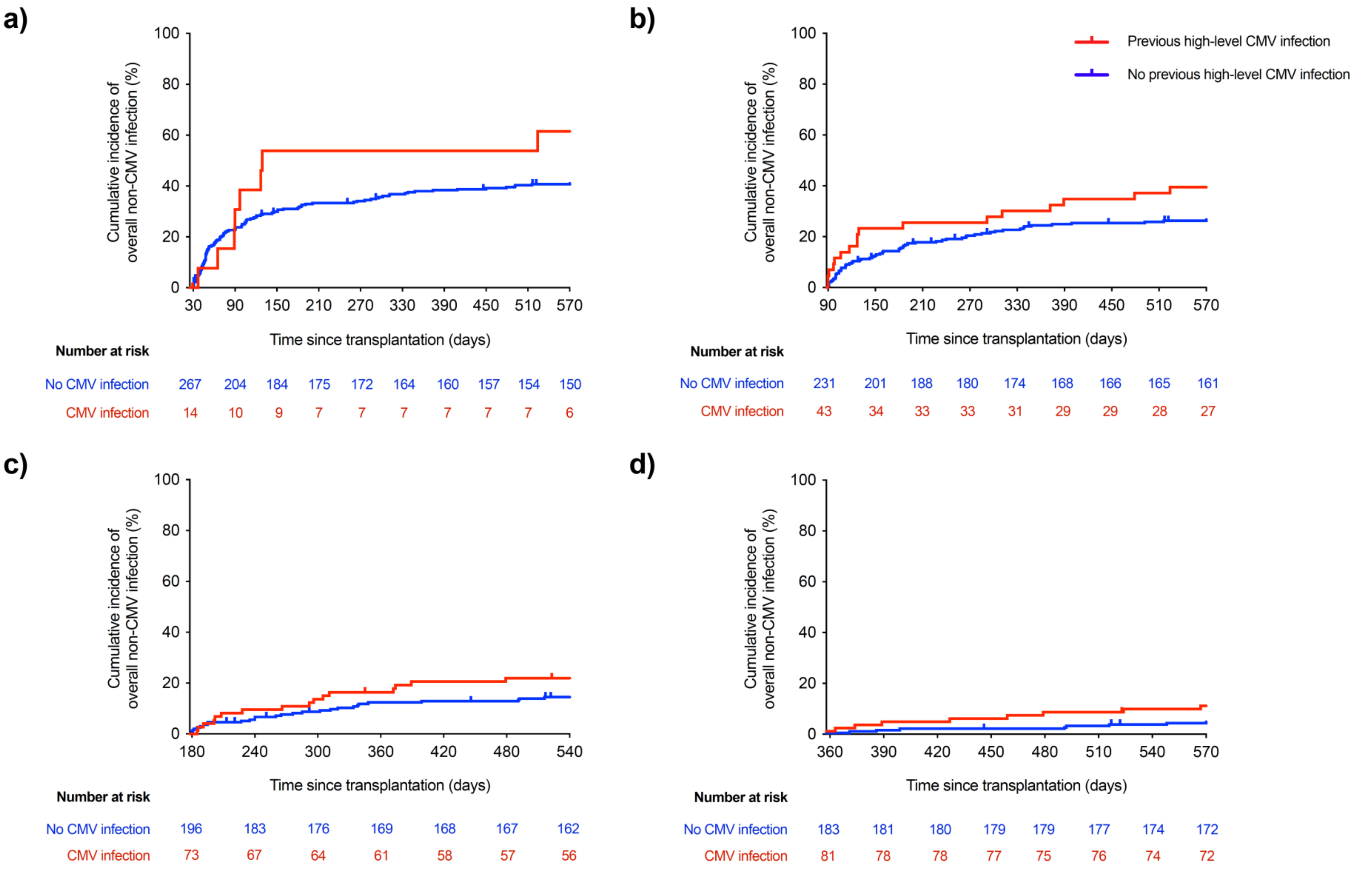

Figure S2. Kaplan-Meier curves for the incidence of overall non-CMV infection according to the recent exposure to CMV infection at any level during the preceding period beyond day 90 (log-rank *P*-value = 0.469) **(a)**, day 180 (log-rank *P*-value = 0.367) **(b)**, and day 360 (log-rank *P*-value = 0.221) **(c)** after transplantation.

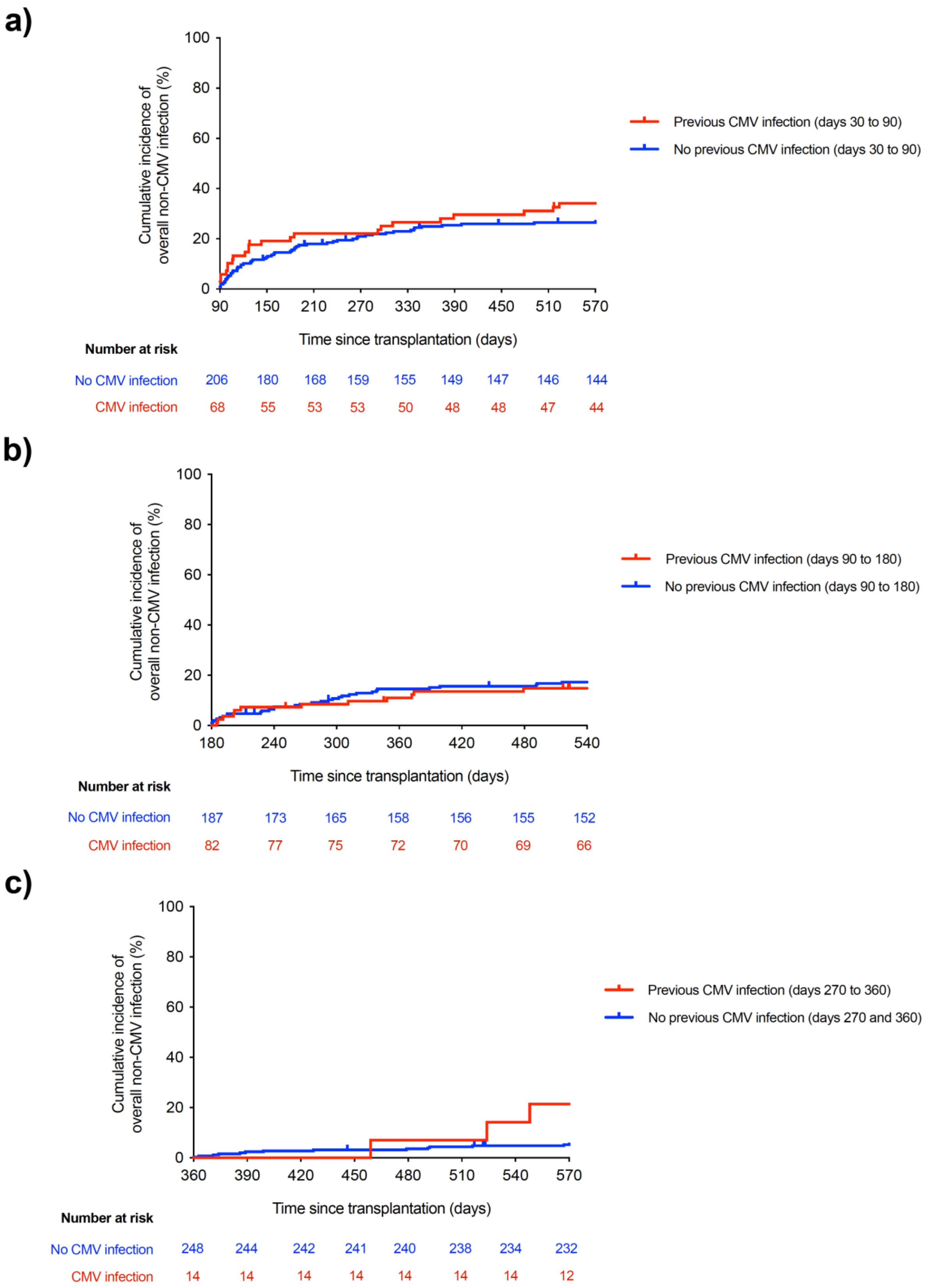

Figure S3. Adjusted hazard ratios (circles) with 95% confidence intervals (whiskers) in landmark Cox regression models for the occurrence of overall non-CMV infection according to the recent exposure to any level or high-level CMV (≥1,000 IU/mL) infection during the preceding period: **(a)** entire study cohort; **(b)** patients not receiving CMV antiviral prophylaxis. Clinical covariates adjusted for are detailed in Table S1. The models beyond day 360 in the group with no antiviral prophylaxis could not constructed due to the low number of patients. Significant associations are depicted in black. CMV: cytomegalovirus.

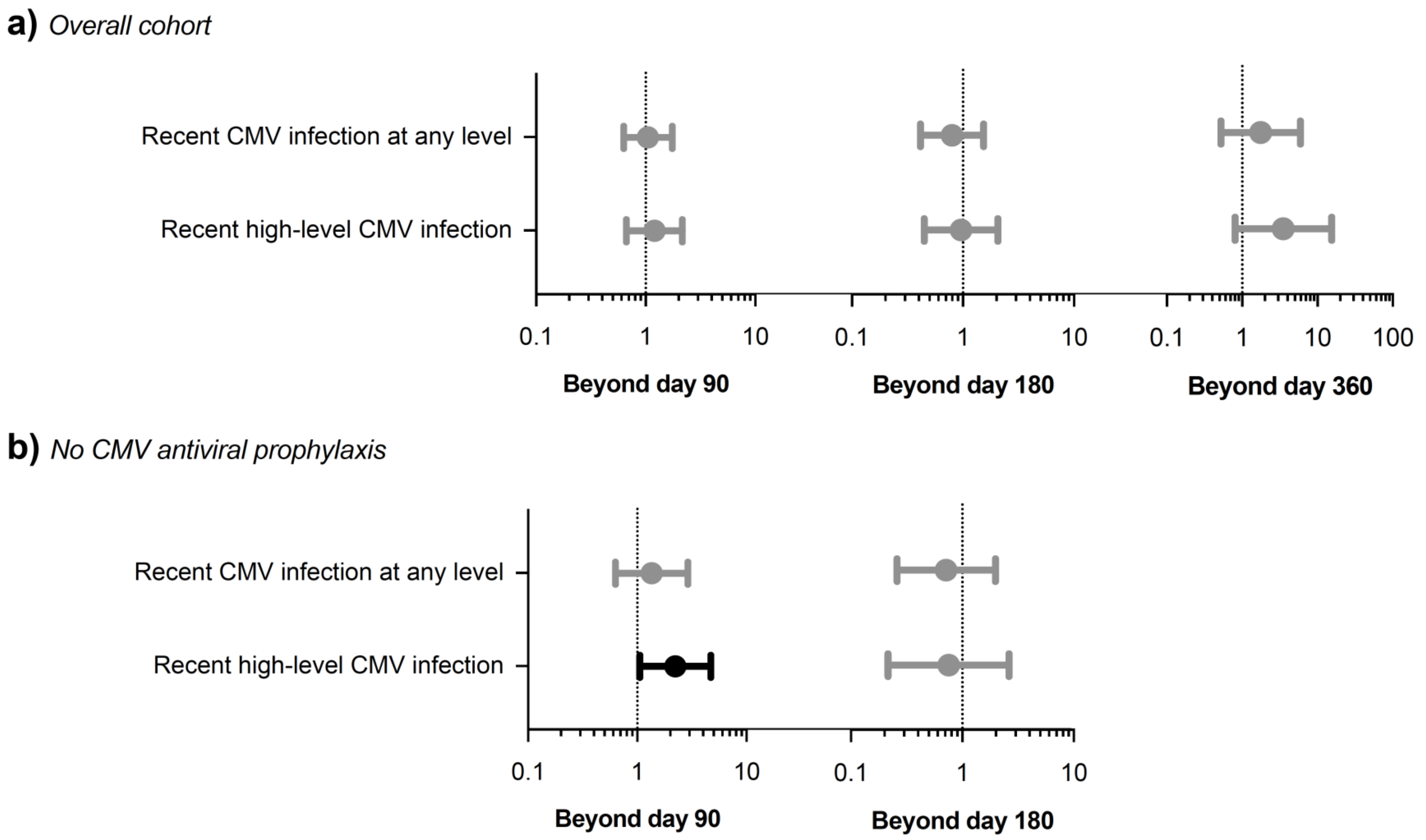

Figure S4. Kaplan-Meier curves for the incidence of bacterial infection (secondary outcome) according to the previous exposure to CMV infection at any level beyond day 30 (log-rank *P*-value = 0.344) **(a)**, day 90 (log-rank *P*-value = 0.679) **(b)**, day 180 (log-rank *P*-value = 0.359) **(c)**, and day 360 (log-rank *P*-value = 0.853) **(d)** after transplantation. CMV: cytomegalovirus.

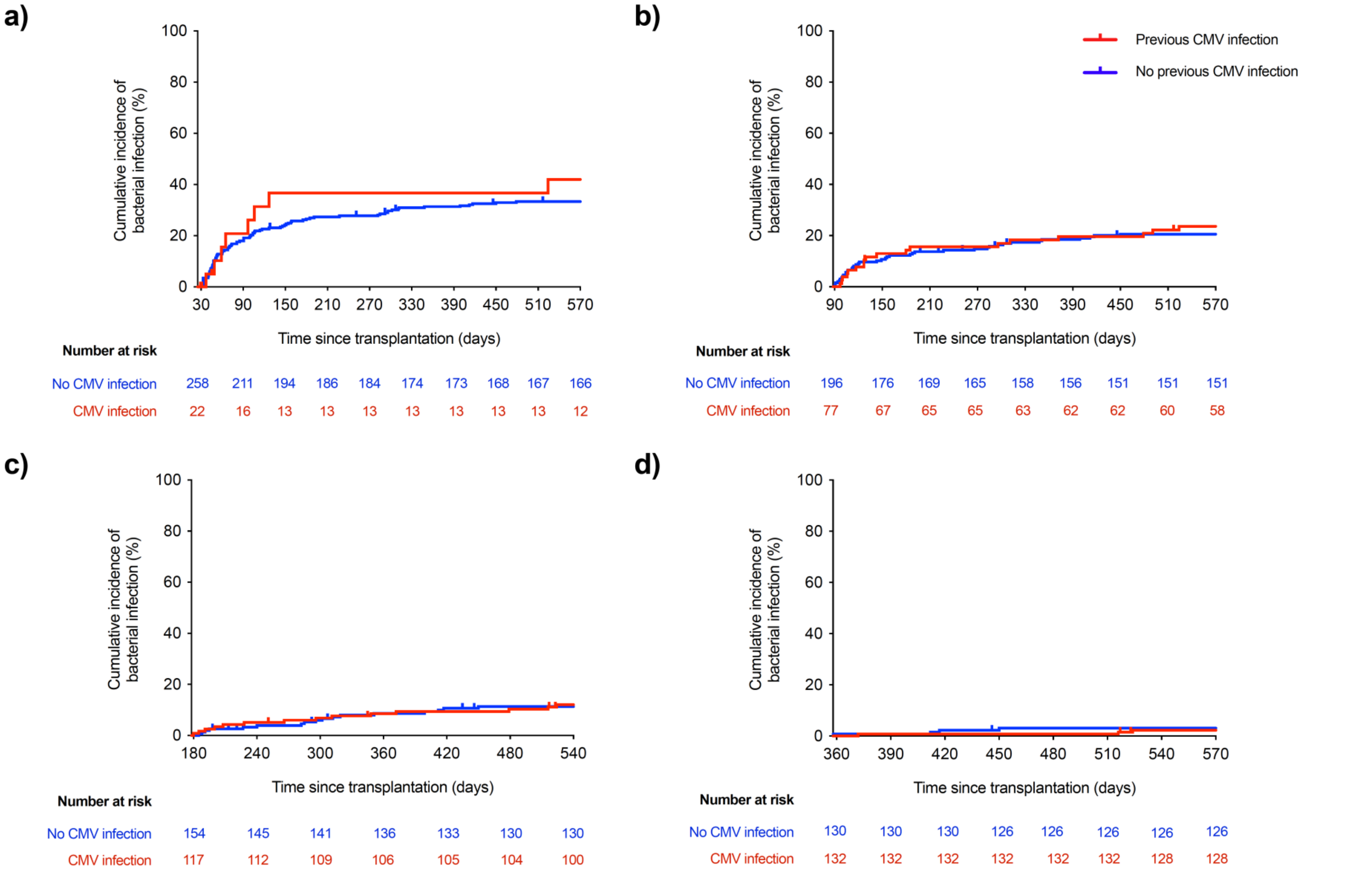

Figure S5. Kaplan-Meier curves for the incidence of opportunistic infection (secondary outcome) according to the previous exposure to CMV infection at any level beyond day 30 (log-rank *P*-value = 0.501) **(a)**, day 90 (log-rank *P*-value = 0.653) **(b)**, day 180 (log-rank *P*-value = 0.629) **(c)**, and day 360 (log-rank *P*-value = 0.497) **(d)** after transplantation. CMV: cytomegalovirus.

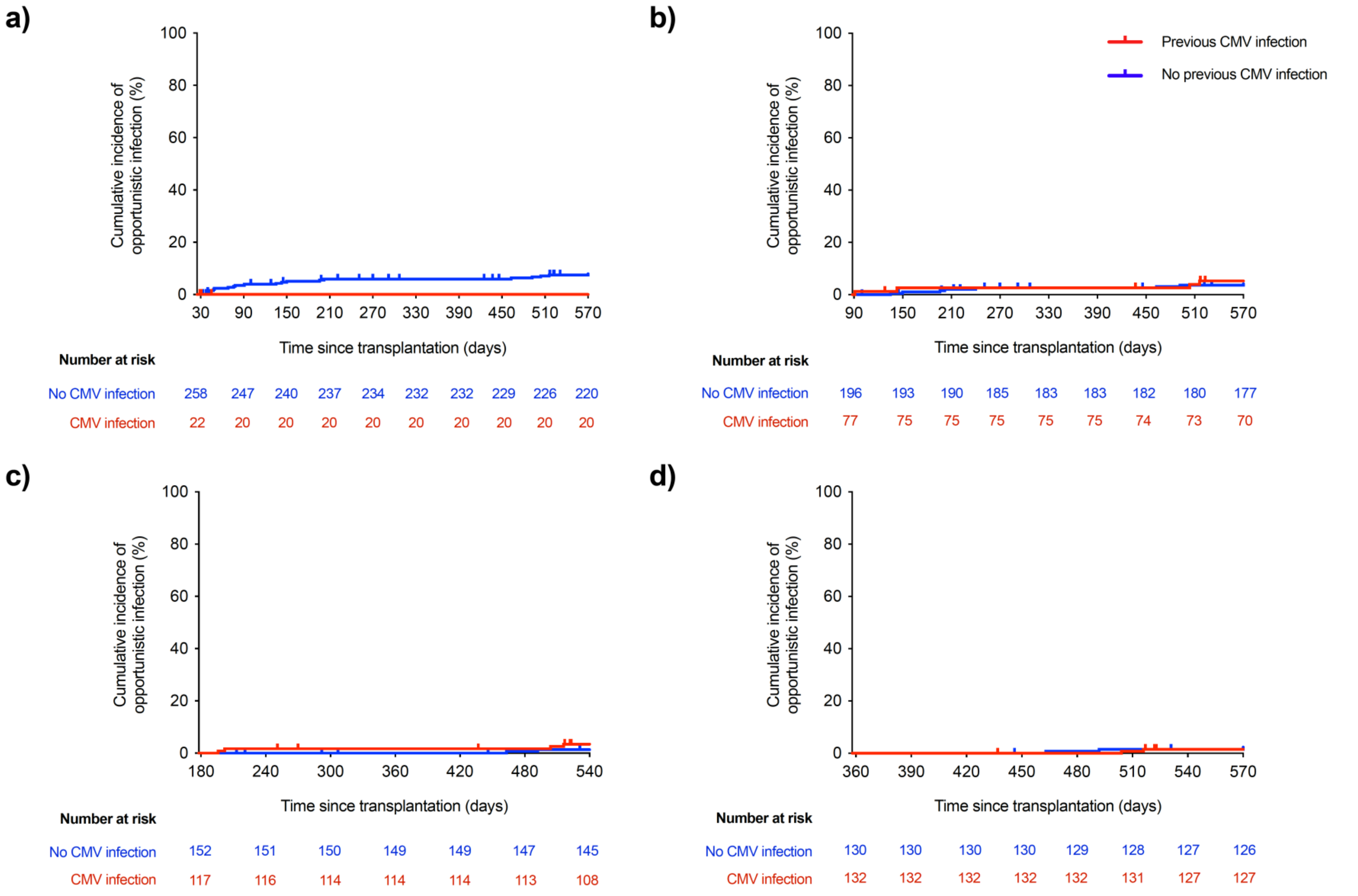

Supplement: Supplementary file 1 [file DataSheet1.pdf]
